# Prevalence of metabolic syndrome in Nepalese type 2 diabetic patients according to WHO, NCEP ATP III, IDF and Harmonized criteria

**DOI:** 10.1186/s40200-014-0104-3

**Published:** 2014-11-23

**Authors:** Daya Ram Pokharel, Dipendra Khadka, Manoj Sigdel, Naval Kishor Yadav, Shreedhar Acharya, Ram Chandra Kafle, Pramod Shankar Shukla

**Affiliations:** Department of Biochemistry, Manipal College of Medical Sciences and Teaching Hospital, Pokhara, Nepal; Department of Internal Medicine, Manipal College of Medical Sciences and Teaching Hospital, Pokhara, Nepal; Department of Laboratory Medicine, Gandaki Medical College Teaching Hospital and Research Center, Prithvi Chowk, Pokhara Nepal; Department of Planning and Research, Cambrian College of Arts and Technology, 1400 Barrydowne Road, Sudbury, ON P3A 3 V8 Canada

**Keywords:** Prevalence, Metabolic syndrome, Type 2 diabetes mellitus, Nepal, Pokhara, Manipal Teaching Hospital

## Abstract

**Background:**

Metabolic syndrome (MetS) present in type 2 diabetic patients greatly increases the risk of strokes and cardiovascular diseases. Timely detection and mapping of MetS facilitates appropriate preventive and therapeutic approaches to minimize these risks. Our study aimed to determine the prevalence of MetS among Nepalese type 2 diabetic patients using WHO (1999), NCEP ATP III (2001), IDF (2005) and Harmonized (2009) definitions and identify the diagnostic concordance and disparity resulting from these four definitions.

**Methods:**

Clinical and biochemical data were collected for 1061 type 2 diabetic patients at Manipal Teaching Hospital, Pokhara, Nepal. The data was analyzed in order to identify prevalence of MetS in these patients. Statistical analysis included usage of Student’s t- and Chi-square tests, kappa statistics and 95% confidence intervals.

**Results:**

The total age adjusted prevalence rates of MetS were 80.3%, 73.9%, 69.9% and 66.8% according to Harmonized, NCEP ATP III, WHO and IDF definitions, respectively. Prevalence increased with the age and was higher in females (p <0.001) according to WHO, NCEP ATP III and Harmonized definitions. Patients of Dalit community had the highest prevalence (p<0.05) according to NCEP ATP III and Harmonized definitions while Mongoloid and Newar patients had the highest prevalence (p <0.05) according to WHO and IDF definitions, respectively. Prevalence was also highest among patient engaged in agriculture occupation. Central obesity and hypertension were respectively the most and the least prevalent components of MetS. The highest overall agreement was between Harmonized and NCEP ATP III definitions (κ =0.62, substantial) and the lowest between WHO & IDF definitions (κ=0.26, slight). The Harmonized definition had the highest sensitivity (99.9%) and negative predictive value (98.9%) while NCEP ATP III definition had the highest specificity (98.9%) and positive predictive values (99.9%) in identifying the cases of MetS.

**Conclusions:**

The prevalence of MetS among Nepalese type 2 diabetic patients was very high suggesting that these patients were at increased risk of strokes, cardiovascular diseases and premature death. The Harmonized definition was the most sensitive while NCEP ATP III and IDF definitions were the most specific in detecting the presence of MetS in Nepalese type 2 diabetic patients.

## Background

Metabolic syndrome (MetS) is a cluster of interconnected metabolic disorders that includes insulin resistance, dysglycemia, central obesity, high triglycerides, low high density lipoprotein cholesterol and hypertension [1]. Recent inclusion of additional metabolic disorders such as chronic pro-inflammatory and prothrombotic states, non-alcoholic fatty liver disease and sleep apnea has made this definition even more complex. Existence of three or more of these disorders warrants the diagnosis of this syndrome. Metabolic syndrome has been shown to increase the risk of cardiovascular diseases (CVDs) by two fold and type 2 diabetes mellitus (DM) approximately by five folds over 5 to 10 years [2-4].

There is still lack of clearly defined pathophysiology and universal definition of MetS. Many researchers question its own existence as a specific syndrome and believe that it is instead a surrogate of combined syndrome that predisposes an individual to particular risk. This has led to several definitions for MetS being proposed by various international regulatory bodies [1]. World Health Organization (WHO) defines this syndrome as the presence of glucose intolerance or insulin resistance or diabetes mellitus with any two of the following components: obesity, high serum triglycerides, low serum high density lipoprotein cholesterol and hypertension [5]. The National Cholesterol Education Program Adult Treatment Panel III (NCEP ATP III) describes metabolic syndrome as the presence of any three of the following components: abdominal obesity, dyslipidemia (high levels of triglycerides, low HDL), hypertension, and elevated fasting glucose [6]. The International Diabetes Federation (IDF) takes central obesity as a mandatory component for the diagnosis of MetS along with any two of the other components: hypertension, abnormal blood glucose, high serum triglycerides and low high density lipoprotein cholesterol [7]. Recently, IDF, National Heart, Lung and Blood Institute (NHLBI), American Heart Association (AHA), World Heart Federation (WHF), International Atherosclerosis Society (IAS) and International Association for the Study of Obesity (IASO) have proposed a new harmonized definition which requires any three of the five components included in the IDF definition for the diagnosis of MetS and do not consider central obesity as an obligatory component [8].

Prevalence of type 2 diabetes mellitus is increasing very rapidly, particularly in developing countries of the world resulting in a substantial burden on the healthcare services [9]. Approximately 9.5% of the Nepalese population suffers from the type 2 diabetes mellitus [10]. Majority of the type 2 diabetic patients also have MetS and are predisposed to higher risk of cardiovascular diseases, strokes and premature death compared to both non-diabetic individuals and diabetic individuals without MetS [11-13]. Presence of MetS in the type 2 diabetic patients has been shown to decrease the survival rate at least by 10 years [14]. Despite controversies on its own existence and universally accepted definition, metabolic syndrome is still a useful concept which helps identify diabetic patients at high risk of developing atherosclerotic CVDs and stroke and predict all cause mortality [1,14]. Moreover, it also helps clinical researchers better understand the pathophysiology that culminates in the CVDs and stroke and formulate preventive and therapeutic approaches. Except for a few preliminary studies [15-18], there has been no systematic study in Nepal that determines the prevalence of MetS in type 2 diabetic populations and diagnostic performance of the available defining criteria. The aim of this study is therefore to estimate the prevalence of MetS in type 2 diabetic patients using four most popular diagnostic criteria viz. WHO (1999), NCEP ATP III (2001), IDF (2005) and Harmonized (2009) (Table 1) and determine their level of agreement and disparity in the diagnosis of MetS. The results of this study will provide template epidemiological data for conducting nationwide prevalence surveys, formulation of national strategies for the prevention and control of MetS, type 2 diabetes and CVDs in Nepal.

**Table 1:** **Criteria for clinical diagnosis of the metabolic syndrome (MetS) according to various definitions**

**Criteria**	**WHO (1998)**	**NCEP (2001)**	**IDF (2005)**	**Harmonized (2009)**
Prerequisite	DM, IFG, IGT, IR	None	WC: ≥90 cm (men) & ≥80 cm (women)†	None
No. of other criteria	and ≥2 of:	≥3 of:	and ≥2 of:	≥3 of:
Obesity	BMI: ≥30 &/or WHR: >0.9 (men) & >0.85 (women)	WC: ≥102 cm (men) & ≥88 cm (women)	Already considered as perquisite criterion	WC: ≥90 cm (men) & ≥80 cm (women)†
BP (mmHg)	≥140/90	≥130/85 or Rx	≥130/85 or Rx	≥130/85 or Rx
HDL-C (mg/dl)	<35 (men) & <39 (women) or	<40 (men) & <50 (women) or Rx	<40 (men) & <50 (women) or Rx	<40 (men) & <50 (women) or Rx
TG (mg/dl)	≥150	≥150 or Rx	≥150 or Rx	≥150 or Rx
Fasting glucose (mg/dl)	≥110, IGT	≥100 or Rx	≥100 or Rx	≥100 or Rx
Microalbuminuria	Urinary albumin ≥20 μg/min or albumin-creatinine ratio >30 mg/g	-	-	-

†Recommended waist circumference thresholds for the abdominal obesity in people of Asian origin.

## Methods

### Study design and patients

This was a cross-sectional study conducted from July 2012 to August, 2013 at Manipal Teaching Hospital (MTH), Pokhara, Nepal. The study protocol was approved by the institutional ethical committee and informed consent was obtained from all the enrolled study patients for their inclusion in the screening and participation in the research. A total of 1061 type 2 diabetic patients without any diabetes related complications and other acute or chronic illness were selected from the various out-patient departments of MTH and enrolled for this study. Presence of type 2 diabetes mellitus was ascertained clinically based on the criteria defined by WHO for diabetes mellitus, age of onset of diabetes and types of medications being prescribed [19]. Patients with established cardiovascular diseases, thyroid dysfunction, excessive alcohol or other drug abuse, current psychiatric treatment and current or recent (up to 4 months) pregnancy were excluded to homogenize the study subjects. The subjects were 30–89 years old and hailed mainly from Gandaki, Dhaulagiri, Lumbini and other adjoining zones of the Western Development Region of Nepal.

### Anthropometric, physiological and lifestyle related variables

All the study patients were personally interviewed by the trained interviewers within the hospital premises using a pre-validated set of questionnaire and details of their demographics, clinical and family history, smoking and dietary habits, ethnicity and profession were recorded. Height, weight, waist and hip circumferences of these patients were measured following the standard protocols and were used for calculating their BMI and WHR. Recent WHO guideline for South Asian population (18.5-22.9 kg/m2) was followed to classify their BMI status [20]. Blood pressure was measured in triplicate while on the sitting position using digital sphygmomanometer (TaiDoc Technology Corporation, Taiwan).

### Biochemical investigations in the blood samples

Five ml of fasting venous blood sample was drawn from each subject and then divided into fluoride-oxalate vials, EDTA vacutainers and plain test tubes. Plasma fasting glucose was measured by glucose oxidase/peroxidase method using blood collected in fluoride-oxalate vials. Glycated hemoglobin (HbA_1_C) was measured on EDTA blood by ion-exchange resin method. Serum triglycerides (TG), total cholesterol (TC) and HDL-cholesterol (HDL-C) were directly measured on plain blood and then LDL-cholesterol value was calculated using Friedwald formula [21]. All these parameters were analyzed at the Clinical Biochemistry laboratory of MTH using semi-automated chemistry analyzer (Humalyzer-3500) and ready-to-use reagent kits according to the protocols provided by the manufacturer (Human diagnostics, Germany). For serum lipid reference level, NCEP ATP III guideline was referred [6]. According to this, hypercholesterolemia was defined as TC >200 mg/dl, high LDL-C when value >100 mg/dl, hypertriglyceridemia as TG >150 mg/dl and low HDL-C when value <40 mg/dl. Dyslipidemia was defined by the presence of one or more abnormal serum lipid concentration.

### Statistical analysis

Statistical analysis was performed by SPSS, version 17.0 for Windows (SPSS, IL, Chicago, USA). Data for categorical variables are expressed either in number and percentage (N,%) or percentage and 95% confidence intervals (95% CI). 95% CIs for crude and age standardized rates were calculated according to the modified Wald method and Keyfitz formulas, respectively [22,23]. Standard error values of age standardized rate were calculated by binomial approximation. Numerical data for continuous variables were expressed in the form of mean ± standard deviation. The age-standardized prevalence rates were calculated with the direct method, using the standard population of Nepal estimated by National Population and Housing Census in 2011 [24]. The agreements among the definitions of WHO, NCEP ATP III, IDF and harmonized criteria were assessed with kappa statistics. The level of agreement was categorized as poor with κ ≤0.20, fair with κ =0.21 to 0.40, moderate with κ =0.41 to 0.60, substantial with κ =0.61 to 0.80, and very good with κ >0.80 [25]. Pearson’s Chi-square test (asymp.sig, 2 sided) and Independent sample test (p values, 2 tailed) were used for checking the statistical significance of difference between the proportion and mean values of two or more groups of variables respectively. The tests were considered statistically significant when p <0.05.

## Results

### Baseline characteristics of the study patients

The frequency analysis of socio-demographic, anthropometric, clinical and biochemical parameters of study patients are given in Table 2. A total of 1061 type 2 diabetic patients (male: 589 and female: 472) were enrolled in the present study, with a male to female ratio of about 1.25:1. The mean age of the patients was 56.4 ±10 years and the durations of type 2 diabetes and hypertension were 6.0 ±4.6 and 3.8 ±5.5 years respectively with no significant difference (p >0.05) between males and females. Their ethnic backgrounds were Brahmin (261, 24.6%), Chhetri (253, 23.8%), Dalit (102, 9.6%), Mongol (348, 32.8%) and Newar (97, 9.1%). Seven hundred forty nine (70.6%) patients were from urban areas and 312 (29.9%) were from villages. Their major occupations were agriculture (509, 48.0%), business (318, 30.0%) and office jobs (234, 22.1%). All subjects were under medication for diabetes mellitus, out of which 524 (49.4%) were also undertaking treatment for hypertension. In terms of BMI statuses, thirty (2.8%) individuals were underweight, 306 (28.8%) were at high risk group, 255 (24.0%) were obese type I and 33 (3.1%) were obese type II. Waist circumference was increased in 305 (28.7%) and substantially increased in 483 (45.5%) subjects. The waist-hip ratio was increased in 861 (81.1%) subjects among which 664 (62.6%) had android and 347 (32.7%) had gyenoid type central obesity. There were 125 (11.8%) vegetarian, 936 (88.2%) non-vegetarian, 184 (17.3%), smoker, 205 (19.3%), ex-smoker and 672 (63.3%) non-smoker patients. 1050 (99.0%) patients had good glycemic control while only 11 (1.0%) patients had suboptimal glycemic control. Serum triglycerides were borderline high in 303 (28.6%), high in 373 (35.2%) and very high in 24 (2.3%) subjects. Likewise, serum total cholesterol level was borderline high in 323 (30.4%) and very high in 373 (35.2%) subjects. Serum HDL cholesterol was lower than normal in 842 (79.4%) subjects. There were 263 (24.8%) subjects in pre-hypertensive stage, 343 (32.3%) in hypertensive stage I and 212 (20.0%) in hypertensive stage II and 524 (49.4%) patients were taking medicine for hypertension. The proportion of these parameters differed significantly (p <0.010) between male and female subjects.

**Table 2:** **Frequency analysis of socio-demographic and anthropometric parameters of the study subjects**

**Characteristic variables**	**Male**	**Female**	p-value*	**Total**
n (%)	589 (55.5)	472 (44.5)		1061 (100)
Ethnic groups				
Brahmin	145 (24.6)	116 (24.6)	<0.010	261 (24.6)
Chhetri	163 (27.7)	90 (19.1)		253 (23.8)
Dalit	57 (9.7)	45 (9.5)		102 (9.6)
Mongol	169 (28.7)	179 (37.9)		348 (32.8)
Newars	55 (9.3)	42 (8.9)		97 (9.1)
Occupation				
Agriculture	164 (27.8)	345 (73.1)	<0.001	509 (48.0)
Business	251 (42.6)	67 (14.2)		318 (30.0)
Office job	174 (29.5)	60 (12.7)		234 (22.1)
Place of residence				
Urban	428 (72.7)	321 (68.0)	<0.001	749 (70.6)
Village	161 (27.3)	151 (32.0)		312 (29.4)
Smoking habit				
Non-smokers	338 (54.7)	334 (70.8)		672 (63.3)
Current smokers	108 (18.3)	76 (16.1)	<0.001	184 (17.3)
Ex-smokers	143 (24.3)	62 (13.1)		205 (19.3)
Dietary habit				
Vegetarian	62 (10.5)	63 (13.3)	<0.001	125 (11.8)
Non-vegetarian	527 (89.5)	409 (86.7)		936 (88.2)
General obesity	127 (21.6)	161 (34.1)	<0.001	288 (27.1)
Central obesity				
Android	448 (76.1)	216 (45.8)	<0.001	664 (62.6)
Gyenoid	111 (18.8)	236 (50.0)		347 (32.7)
Glycemic control				
Good (≤6.5%)	589 (100.0)	461 (97.7)	<0.001	1050 (99.0)
Suboptimal (≥6.5%)	0 (0)	11 (2.3)		11 (1.0)
Dyslipidemia				
High TG	377 (64.0)	323 (68.4)	<0.001	700 (66.0)
Low HDL-C	464 (78.8)	378 (80.1)		842 (79.4)
Hypertension (HTN)	410 (52.4)	357 (52.1)	<0.001	555 (32.3)
Treatment for HTN	281 (47.7)	243 (51.5)	<0.001	524 (49.4)

Values are presented as n (%). *p-values (2-tailed): Chi-square test was performed to compare the proportions of selected demographic, anthropometric and biochemical characteristics between male and female diabetic patients.

The mean values of various anthropometric, clinical and biochemical parameters of the male and female study patients are presented in Table 3. When compared to male patients, female patients had significantly higher mean values of BMI, waist circumference, estimated average glucose, HbA1c, triglycerides, very low density lipoprotein, high density lipoprotein cholesterol and diastolic blood pressure (p <0.010). Only the waist to hip ratio was significantly higher (p <0.001) in males compared to females. The gender wise difference was not statistically significant for other parameters (p >0.05).

**Table 3:** **Anthropometric, biochemical and clinical parameters of the type 2 diabetic patients**

**Characteristic variables**	**Male**	**Female**	p-value*	**Total**
n	589	472	-	1061
Age (years)	56.4 ±9.6	56.4 ±10.4	>0.050	56.4 ±10.0
BMI (m/kg^2^)	23.2 ±2.7	24.1 ±2.8	<0.001	23.6 ±2.8
WC (cm)	95.0 ±8.5	96.7 ±10.1	<0.010	95.8 ±9.3
WHR	1.1 ±0.1	1.0 ±0.1	<0.001	1.0 ±0.1
FPG (mg/dl)	132.2 ±37.6	129.8 ±28.3	>0.05	131.1 ±33.8
2 hr PMG (mg/dl)	217.2 ±70.7	219.1 ±50.3	>0.05	218.1 ±62.4
EAG (mg/dl)	131.1 ±19.3	135.8 ±22.7	<0.001	133.2 ±21.0
HbA1C (%)	6.2 ±0.7	6.4 ±0.8	<0.001	6.3 ±0.7
Duration of DM (year)	5.8 ±4.7	6.3 ±4.6	>0.050	6.0 ±4.6
TG (mg/dl)	188.3 ±117.4	229.6 ±236.7	<0.010	206.7 ±181.6
TC (mg/dl)	227.4 ±59.1	231.3 ±74.5	>0.050	229.1 ±66.4
VLDL (mg/dl)	37.7 ±23.4	45.8 ±27.4	<0.010	41.3 ±36.3
HDL-C (mg/dl)	31.9 ±7.6	30.7 ±8.2	<0.050	31.4 ±7.9
LDL-C (mg/dl)	157.8 ±63.7	154.8 ±55.7	>0.050	156.5 ±60.2
SBP (mmHg)	132.4 ±13.8	132.6 ±17.4	>0.050	132.5 ±15.5
DBP (mmHg)	83.4 ±7.7	81.9 ±9.9	<0.010	82.8 ±8.8
Duration of HTN (year)	3.6 ±4.9	4.2 ±6.2	>0.050	3.8 ±5.5

Values are presented as mean ± standard deviation. *p-values (2-tailed): Student’s *t*-test was used to compare the mean values of selected biochemical and anthropometric characteristics between male and female diabetic patients.

### Crude, sex and age-adjusted prevalence of metabolic syndrome

Both age and sex-specific crude and age adjusted prevalence of MetS estimated by using WHO, NCEP ATP III, IDF and Harmonized criteria are given in the Table 4. Most patients met the criteria for metabolic syndrome, and the proportion of patients without metabolic syndrome was relatively small. The total crude prevalence was 81.1%, 83.0%, 80.5% and 91.6% according to WHO, NCEP ATP III, IDF and Harmonized criteria, respectively. The corresponding age-adjusted total prevalence was 69.9%, 73.9%, 66.8% and 80.3% respectively. The Harmonized definition outperformed other definitions in diagnosing the cases of MetS. The prevalence was significantly higher in females (p <0.001) than in males except for the one diagnosed by IDF definition. Prevalence generally increased with the increase in age and remained highest in the age range of 50–69 years in both the sexes. The peak prevalence age group, however, was different for males (50–59 years) and females (70–79 years). Prevalence was at the lower end in the age groups of 30–40 and 80–89 years for both the sexes. Total prevalence of metabolic syndrome was higher among patients living in the urban area according to WHO, NCEP ATPIII and Harmonized definitions and in villages according to IDF definition. However, the difference was significant only with WHO estimated prevalence (p <0.002) (Table 5). On the other hand, according to NCEP ATPIII, IDF and Harmonized definitions, prevalence was much higher (p <0.020) among the female patients living in the villages compared to their urban counterparts. Females had also higher prevalence compared to their male counterparts living in the same places except for the WHO defined prevalence in the urban patients. Likewise, Dalit patients had the highest total prevalence (p <0.05) according to NCEP ATPIII and Harmonized definitions while Mongol and Newar patients had the highest (p <0.010) total prevalence according to WHO and IDF definitions respectively. Sex-wise analysis revealed that Mongol and Dalit male patients had the highest (p <0.010) prevalence according to WHO, NCEP ATPIII and Harmonized definitions whereas Dalit female patients had the highest (p <0.001) total prevalence according to all four definitions. Prevalence was also highest among Chhetri and Newar female patients (p <0.001) according to IDF and Harmonized definitions (Table 6). Depending on the definition used, the prevalence of MetS was also found to have relation with occupation of the study patients. Total prevalence was significantly higher (p <0.05) in patients engaged in agricultural activity according to all four definitions and found to have relation with the gender. Male patients engaged in agricultural activity had the highest prevalence according to WHO, NCEP ATPIII and Harmonized definitions while it was highest among female patients involved in business occupation according to NCEP ATPIII and Harmonized definitions and office jobs according to WHO and IDF definitions (Table 7). However, the difference in prevalence among females of different occupations was not statistically significant (p >0.05) except for the one defined by IDF criteria (p <0.001).

**Table 4:** **Age and sex-specific and age-adjusted prevalence of metabolic syndrome in the type 2 diabetic patients**

**Age group (years)**	**Prevalence of MetS (WHO) (%)**			**Prevalence of MetS (NCEP ATPIII) (%)**		
	**Male**	**Female**	*p-value	**Male**	**Female**	*p-value
n	589	472	-	589	472	-
30-39	14 (50.0)	15 (50.0)	>0.050	14 (50.0)	16 (53.3)	>0.050
40-49	88 (67.2)	60 (67.4)	>0.050	103 (78.6)	82 (92.1)	<0.010
50-59	157 (84.4)	148 (95.5)	<0.010	145 (78.0)	147 (94.8)	<0.001
60-69	151 (83.9)	111 (82.2)	>0.050	143 (79.4)	122 (90.4)	<0.010
≥70	61 (95.3)	56 (88.9)	>0.050	57 (89.1)	55 (87.3)	>0.050
Sex-specific crude	471 (80.0)	390 (82.6)	>0.050	462 (78.4)	422 (89.4)	<0.001
Total crude	861 (81.1)			884 (83.3)		
Total age-adjusted	69.9			73.9		
**Age group (years)**	**Prevalence of MetS (IDF) (%)**			**Prevalence of MetS (Harmonized) (%)**		
	**Male**	**Female**	*p-value	**Male**	**Female**	*p-value
n	589	472	-	589	472	-
30-39	4 (14.3)	18 (60.0)	<0.001	14 (50.0)	18 (60.0)	>0.050
40-49	91 (69.5)	77 (86.5)	<0.010	110 (84.0)	87 (97.8)	<0.010
50-59	152 (81.7)	153 (98.7)	<0.001	182 (97.8)	153 (98.7)	>0.050
60-69	121 (67.2)	135 (100)	<0.001	150 (83.3)	135 (100)	<0.001
≥70	37 (63.8)	58 (98.3)	<0.001	57 (98.3)	58 (98.3)	>0.050
Sex-specific crude	410 (69.6)	444 (94.1)	<0.001	518 (87.9)	454 (96.2)	<0.001
Total crude	854 (80.5)			972 (91.6)		
Total age-adjusted	66.8			80.3		

Values are expressed as n (%) or only%. *p-values (2-tailed): Chi square test was used to compare the prevalence of metabolic syndrome between male and female diabetic patients.

**Table 5:** **Prevalence of metabolic syndrome in the type 2 diabetic patients according to their place of residence**

**Defining bodies**	**Sex**	**n**	**Place of residence**		*p-value
			**Urban**	**Village**	
	Total	860	626 (83.5)	234 (75.2)	<0.010
WHO	Male	470	368 (85.8)	102 (63.8)	<0.001
	Female	390	258 (80.4)	132 (87.4)	>0.050
	p-value		<0.050	<0.001	
	Total	884	633 (84.4)	251 (80.7)	>0.050
NCEP ATP III	Male	462	353 (82.3)	109 (68.1)	<0.001
	Female	422	280 (87.2)	142 (94.0)	<0.050
	p-value		>0.050	<0.001	
	Total	854	589 (78.5)	265 (85.2)	<0.050
IDF	Male	410	295 (68.8)	115 (71.9)	>0.050
	Female	444	294 (91.6)	150 (99.3)	<0.010
	p-value		<0.001	<0.001	
	Total	972	687 (91.6)	285 (91.6)	>0.050
Harmonized	Male	518	383 (89.3)	135 (84.4)	>0.050
	Female	454	304 (94.7)	150 (99.3)	<0.050
	p-value		<0.010	<0.001	

Values are presented as n (%). *p-values (2-tailed): Chi-square tests were performed to compare the prevalence of MetS between male and female patients residing in urban and village areas (rows) and within the individual place of residence (column).

**Table 6:** **Prevalence of metabolic syndrome in the type 2 diabetic patients according to their ethnic background**

			**Ethnic groups**					
**Defining bodies**	**Sex**	**n**	**Brahman**	**Chhetri**	**Mongol**	**Dalit**	**Newar**	*p-value
WHO	Total	860	181 (69.3)	187 (73.9)	331 (95.1)	92 (90.2)	69 (71.1)	<0.001
	Male	470	124 (85.5)	109 (66.9)	159 (94.1)	47 (82.5)	31 (56.4)	<0.001
	Female	390	57 (49.1)	78 (86.7)	172 (96.1)	45 (100.0)	38 (90.5)	<0.001
	*p-value		<0.001	<0.010	>0.050	<0.010	<0.001	
NCEP ATPIII	Total	884	204 (78.2)	196 (77.5)	309 (88.5)	98 (96.1)	77 (79.4)	<0.010
	Male	462	109 (75.2)	117 (71.8)	141 (83.4)	55 (96.5)	40 (72.7)	<0.010
	Female	422	95 (81.9)	79 (87.8)	168 (93.9)	43 (95.6)	37 (88.1)	<0.050
	p-value		>0.050	<0.010	<0.010	>0.050	>0.050	
IDF	Total	854	178 (68.2)	214 (84.6)	296 (85.1)	82 (80.4)	84 (86.6)	<0.001
	Male	410	87 (60.0)	124 (76.1)	120 (71.0)	37 (64.9)	42 (76.4)	<0.050
	Female	444	91 (78.4)	90 (100.0)	176 (98.3)	45 (100.0)	42 (100.0)	<0.001
	p-value		<0.010	<0.001	<0.001	<0.001	<0.010	
Harmonized	Total	972	217 (83.1)	233 (92.1)	336 (96.6)	102 (100.0)	84 (86.6)	<0.001
	Male	518	116 (80.0)	143 (87.7)	160 (94.7)	57 (100.0)	42 (76.4)	<0.001
	Female	454	101 ((87.1)	90 (100.0)	176 (98.3)	45 (100.0)	42 (100.0)	<0.001
	p-value		>0.050	<0.010	>0.050	^ǂ^	<0.010	

Values are presented as n (%). *p-values (2-tailed): Chi-square tests were performed to compare the prevalence of MetS between the male and female patients of different ethnic groups (rows) and within the individual ethnic groups (column). ^ǂ^No statistics were computed because metabolic syndrome defined by Harmonized definition is a constant.

**Table 7:** **Prevalence of metabolic syndrome in the type 2 diabetic patients according to their occupations**

**Defining bodies**	**Sex**	**n**	**Agriculture**	**Business**	**Office job**	*p-value
**WHO**	Total	860	422 (82.9)	260 (81.8)	178 (76.1)	>0.050
	Male	470	137 (83.5)	206 (82.1)	127 (73.0)	<0.050
	Female	390	285 (82.6)	54 (80.6)	51 (85.0)	>0.050
	p-value	-	>0.050	>0.050	>0.050	-
**NCEP ATPIII**	Total	884	447 (87.8)	241 (75.8)	196 (83.8)	<0.001
	Male	462	141 (86.0)	177 (70.5)	144 (82.8)	<0.001
	Female	422	306 (88.7)	64(95.5)	52 (86.7)	>0.050
	p-value	-	>0.050	<0.001	>0.050	-
**IDF**	Total	854	439(86.2)	236(74.2)	179 (76.5)	<0.001
	Male	410	109 (66.5)	180 (71.7)	121 (69.5)	>0.050
	Female	444	330 (95.7)	56 (83.6)	58 (96.7)	<0.001
	p-value	-	<0.001	<0.050	<0.001	-
**Harmonized**	Total	972	479 (94.1)	286 (89.9)	207 (88.5)	<0.050
	Male	518	149 (90.9)	220 (87.6)	149 (85.6)	>0.050
	Female	454	330 (95.7)	66 (98.5)	58 (96.7)	>0.050
	p-value	-	<0.050	<0.010	<0.050	-

Values are presented as n (%). *p-values (2-tailed): Chi-square tests were performed to compare the prevalence of MetS between male and female patients of different occupations (rows) and within the individual occupation (column).

### Prevalence of individual components of metabolic syndrome

The frequencies of the number of MetS components present in male and female patients are summarized in Table 8. Majority of the Subjects had the cluster of four metabolic abnormalities and the overall prevalence differed significantly (p <0.05) between male and females. The prevalence of individual MetS components included in the WHO, NCEP ATPIII, IDF and Harmonized criteria is shown in Table 9. The most prevalent component was the central obesity according to WHO (98.8%) and IDF (99.9%) definitions. Decreased HDL-cholesterol was the second most prevalent component according to NCEP ATPIII (95.2%) and Harmonized (94.2%) definitions. Increased BMI (≥30 kg/m^2^) was the least prevalent component (4.0%) according to WHO definition while hypertension was the least frequent component by the NCEP ATPIII (72.1%), IDF (65.9%) and Harmonized criteria (67.0%).

**Table 8:** **Frequency of individual components of metabolic syndrome in the type 2 diabetic patients**

**MetS components**	**WHO (1998)**		**NCEP ATP (2001)**		**IDF (2005)**		**Harmonised (2009)**	
	**Male**	Female*	**Male**	Female**	**Male**	Female**	**Male**	Female**
n	589	472	589	472	589	472	589	472
1	6 (1.0)	1 (0.2)	25 (4.2)	4 (.8)	70 (11.9)	19 (4.0)	70 (11.9)	19 (4.0)
2	102 (17.3)	82 (17.4)	102 (17.3)	46 (9.7)	129 (21.9)	71 (15.0)	128 (21.7)	71 (15.0)
3	230 (39.0)	149 (31.6)	179 (30.4)	65 (13.8)	226 (38.4)	166 (35.2)	227 (38.5)	166 (35.2)
4	251 (42.6)	240 (50.8)	222 (37.7)	152 (32.2)	164 (27.8)	216 (45.8)	164 (27.8)	216 (45.8)
5	-	-	61 (10.4)	205 (43.4)	-	-	-	-

Values are presented as n (%). *p <0.050, **p <0.001 (two sided). Chi-square test was performed to compare the number of components of metabolic syndrome between male and female patients.

**Table 9:** **Prevalence of individual metabolic abnormalities in the type 2 diabetic patients with metabolic syndrome**

	**WHO**				**NCEP ATP III**			
	**Prevalence (%)**		*p-value		**Prevalence (%)**		*p-value	
**Components of MetS**	**Male**	**Female**		**Total**	**Male**	**Female**		**Total**
n	470	386		856	465	429		894
Increased FG^ǂ^	470 (100)	386 (100)	-	856 (100)	465 (100)	429 (100)	-	894 (100)
BMI (≥30 kg/m^2^)	10 (2.1)	24 (6.2)	<0.010	34 (4.0)	-	-		-
Central obesity	460 (97.9)	386 (100)	<0.010	846 (98.8)	132 (28.4)	368 (85.8)	<0.001	500 (55.9)
Increased TG	368 (78.3)	303 (78.5)	>0.050	671 (78.4)	363 (78.1)	324 (75.5)	>0.050	687 (76.8)
Decreased HDL-C	373 (79.4)	344 (89.1)	<0.001	717 (83.8)	425 (91.4)	426 (99.3)	<0.001	851 (95.2)
Hypertension	300 (63.8)	267 (69.2)	>0.050	567 (66.2)	350 (75.3)	295 (68.8)	<0.050	645 (72.1)
	**IDF**				**Harmonized**			
	**Prevalence (%)**		*p-value		**Prevalence (%)**		*p-value	
**Components of MetS**	**Male**	**Female**		**Total**	**Male**	**Female**		**Total**
n	400	434		834	518	454		972
Increased FG^ǂ^	400 (100)	434 (100)	-	834 (100)	518 (100)	454 (100)	-	972 (100)
Central obesity	400 (100)	433 (99.8)	>0.050	833 (99.9)	399 (77.0)	433 (95.4)	<0.001	832 (85.6)
Increased TG	268 (67.0)	304 (68.6)	>0.050	572 (58.2)	367 (70.8)	324 (71.4)	>0.050	691 (71.1)
Decreased HDL-C	349 (87.3)	431 (99.3)	<0.001	780 (93.5)	468 (90.3)	451 (99.3)	<0.001	819 (94.5)
Hypertension	256 (64.0)	286 (65.9)	>0.050	542 (62.0)	355 (68.5)	296 (65.2)	>0.050	651 (67.0)

Values are presented as n (%). ^ǂ^All the study patients were considered to have increased fasting glucose as they were diagnosed type 2 diabetic patients and taking medication for blood glucose control. *p-values (2-tailed): Chi-square test was performed to compare the mean proportions of metabolic components between male and female patients.

### Comparison between groups with and without metabolic syndrome

The subjects with metabolic syndrome were comparatively older, more overweight or obese, hyperglycemic, insulin resistant and suffering from diabetes for longer duration. They had relatively poor glycemic control, increased serum triglycerides, decreased serum HDL-cholesterol and hypertension of at least four year duration. BMI, waist circumference, HbA1C, serum triglycerides and number of metabolic components were significantly higher (p <0.05) in males with MetS whereas waist-hip ratio and insulin resistance level were significantly higher (p <0.05) in females with MetS diagnosed by three or more definitions of metabolic syndrome. There was no significant difference (p >0.05) in duration of diabetes and hypertension, systolic and diastolic blood pressure, fasting glucose, insulin and HDL-cholesterol levels between male and female with MetS defined at least by three definitions. Detailed comparison of clinical and biochemical parameters between groups with and without MetS is presented in Table 10.

**Table 10:** **Comparison of anthropometric, biochemical and clinical parameters in the type 2 diabetic patients with and without metabolic syndrome**

	**WHO**			**NCEP ATPIII**		
**Variables**	**With MetS**	**Without MetS**	*p-value	**With MetS**	**Without MetS**	*p-value
(n,%)	861 (81.5)	200 (18.5)	<0.001	884 (83.2)	177 (16.8)	<0.001
Age (year)	57.4 ±9.4	52.1 ±11.2	<0.001	56.9 ±9.5	53.5 ±11.7	<0.001
BMI (kg/m^2^)	23.8 ±2.9	23.0 ±2.1	<0.001	23.7 ±2.9	23.0 ±2.0	<0.001
WC (cm)	96.9 ±9.2	90.9 ±8.1	<0.001	96.8 ±9.3	90.4 ±6.9	<0.001
WHR	1.05 ±0.1	0.98 ±0.1	<0.001	1.04 ± .01	0.99 ±0.09	<0.001
FPG (mg/dl)	134.2 ±35.4	117.9 ±21.1	<0.001	132.9 ±34.9	122.0 ±25.8	<0.001
2 hr PMG (mg/dl)	226.5 ±63.8	182.1 ±39.4	<0.001	224.7 ±63.3	185.0 ±45.5	<0.001
EAG (mg/dl)	137.1 ±20.0	116.3 ±16.8	<0.001	136.5 ±20.1	116.6 ±17.3	<0.001
Hb1Ac%	6.4 ±0.7	5.7 ±0.6	<0.001	6.4 ±0.7	5.7 ±0.6	<0.001
Duration of DM (year)	6.5 ±4.7	4.0 ±3.6	<0.001	6.3 ±4.7	4.6 ±3.7	<0.001
TG (mg/dl)	223.0 ±195.8	136.3 ±61.3	<0.001	222.6 ±190.4	127.1 ±95.0	<0.001
TC (mg/dl)	239.9 ±67.1	183.1 ±37.4	<0.001	239.9 ±66.2	175.4 ±33.0	<0.001
VLDL (mg/dl)	44.5 ±29.2	27.5 ±12.3	<0.001	44.4 ±38.1	25.4 ±18.9	<0.001
HDL-C (mg/dl)	29.5 ±6.7	39.5 ±7.5	<0.001	29.5 ±6.8	40.7 ±6.3	<0.001
LDL-C (mg/dl)	165.9 ±61.0	116.2 ±35.3	<0.001	166.0 ±59.9	109.2 ±34.2	<0.001
SBP (mmHg)	135.2 ±15.0	120.8 ±11.6	<0.001	134.5 ±14.9	122.4 ±14.2	<0.001
DBP (mmHg)	84.1 ±8.8	76.9 ±5.5	<0.001	83.9 ±8.6	76.9 ±7.0	<0.001
Duration of HTN (year)	4.7 ±5.8	0.0 ±0.0	<0.001	4.5 ±5.8	0.6 ±1.9	<0.001
No. of MetS components	3.6 ±0.5	2.0 ±0.3	<0.001	4.0 ±0.8	1.8 ±0.4	<0.001
	**IDF**			**Harmonized**		
**Variables**	**With MetS**	**Without MetS**	*p-value	**With MetS**	**Without MetS**	*p-value
n (%)	854 (80.5)	207 (19.5)	<0.001	972 (91.6)	89 (8.4)	<0.001
Age (year)	57.1 ±9.4	53.5 ±11.8	<0.001	56.9 ±9.5	50.5 ±12.7	<0.001
BMI (kg/m^2^)	24.1 ±2.6	21.6 ±2.2	<0.001	23.7 ±2.8	22.7 ±2.2	<0.001
WC (cm)	98.2 ±8.0	85.6 ±6.7	<0.001	96.4 ±9.2	88.8 ±7.5	<0.001
WHR	1.05 ±0.09	0.96 ±0.08	<0.001	1.0 ±0.1	0.95 ±0.1	<0.001
FPG (mg/dl)	131.2 ±30.8	130.9 ±43.9	>0.050	132.4 ±34.5	117.1 ±20.0	<0.001
2 hr PMG (mg/dl)	220.7 ±57.3	207.2 ±79.3	<0.050	222.6 ±62.6	167.9 ±30.5	<0.001
EAG (mg/dl)	135.9 ±20.6	122.0 ±19.0	<0.001	135.5 ±20.2	108.3 ±12.8	<0.001
Hb1Ac%	6.4 ±0.7	5.9 ±0.7	<0.001	6.3 ±0.7	5.4 ±0.5	<0.001
Duration of DM (year)	6.4 ±4.7	4.3 ±4.0	<0.001	6.2 ±4.7	3.7 ±3.6	<0.001
TG (mg/dl)	217.7 ±99.2	161.2 ±51.8	<0.001	214.3 ±187.7	123.2 ±25.7	<0.001
TC (mg/dl)	233.4 ±68.6	211.7 ±53.2	<0.001	234.3 ±66.4	173.2 ±32.6	<0.001
VLDL (mg/dl)	43.4 ±39.9	32.4 ±10.3	<0.001	42.8 ±37.6	25.1 ±5.5	<0.001
HDL-C (mg/dl)	30.7 ±7.4	34.2 ±9.2	<0.001	30.3 ±7.1	43.1 ±5.8	<0.001
LDL-C (mg/dl)	159.3 ±60.8	145.0 ±56.4	>0.010	161.2 ±60.0	105.0 ±33.7	<0.001
SBP (mmHg)	132.4 ±15.7	132.7 ±14.6	>0.050	132.9 ±15.3	128.3 ±16.9	<0.050
DBP (mmHg)	83.0 ±9.0	81.6 ±7.5	<0.050	83.1 ±8.9	78.7 ±6.7	<0.001
Duration of HTN (year)	4.3 ±5.8	2.0 ±3.5	<0.001	4.1 ±5.7	0.7 ±1.4	<0.001
No. of MetS components	4.2 ±0.7	3.0 ±0.9	<0.001	4.2 ±0.8	2.0 ±0.2	<0.001

Values are expressed as mean ± standard deviation.*p-values (2-tailed): Students *t*-test comparing the mean values of selected characteristics between patients with and without MetS.

### Agreement and disparity among MetS definitions

The agreement and disparity in the diagnosis of MetS among WHO, NCEP ATP III, IDF and Harmonized definitions is presented in the Table 11. The agreement among these four definitions was substantial to slight. The agreement was substantial between NCEP ATP III-Harmonized (κ =0.62 (0.55-0.69), p <0.001), moderate between WHO-NCEP ATPIII (κ =0.51 (0.45-0.58), p <0.001) and IDF-Harmonized (κ =0.51 (0.47-0.61), p <0.001), fair between WHO-Harmonized (κ =0.37 (0.30-0.44), p <0.001) and NCEP ATPII-IDF (κ =0.33 (0.26-0.40), p <0.001) and slight between WHO-IDF definitions (κ =0.27 (0.19-0.33), p <0.001). The Harmonized-NCEP ATPIII and IDF definitions had the highest sensitivity (99.9%) and negative predictive value (98.9%) whereas NCEP ATPIII-Harmonized definitions had the highest specificity (98.9%) and positive predictive value (99.9%).

**Table 11:** **The concordance and diagnostic accuracy of WHO, NCEP ATPIII, IDF and Harmonized definitions in identifying the cases of metabolic syndrome in the type 2 diabetic patients**

	**Concordance**			**Diagnostic Accuracy (%)**			
**Definitions**	**κ-value (95%** **CI)**	*p-value	**Agreement**	**Sensitivity**	**Specificity**	**PPV**	**NPV**
WHO vs NCEP ATPIII	0.53 (0.45-0.58)	<0.001	Moderate	90.0	71.4	92.8	63.5
NCEP ATPIII vs WHO	0.53 (0.45-0.58)	<0.001	Moderate	92.8	57.5	90.4	65.0
WHO vs IDF	0.27 (0.19-0.33)	<0.001	Slight	86.2	39.6	85.5	41.0
IDF vs WHO	0.27 (0.19-0.33)	<0.001	Slight	85.5	41.0	86.2	39.6
WHO vs Harmonized	0.37 (0.30-0.44)	<0.001	Fair	86.0	71.9	97.1	32.0
Harmonized vs WHO	0.37 (0.30-0.44)	<0.001	Fair	97.1	32	86	71.9
NCEP ATPIII vs IDF	0.33 (0.26-0.40)	<0.001	Fair	89.5	42.0	86.4	49.2
IDF vs NCEP ATPIII	0.33 (0.26-0.40)	<0.001	Fair	86.4	49.2	89.5	42.0
NCEP ATPIII vs Harmonized	0.62 (0.55-0.69)	<0.001	Substantial	90.8	98.9	99.9	49.7
Harmonized vs NCEP ATP III	0.62 (0.55-0.69)	<0.001	Substantial	99.9	49.7	90.8	98.9
IDF vs Harmonized	0.54 (0.47-0.61)	<0.001	Moderate	87.8	98.9	99.5	42.5
Harmonized vs. IDF	0.54 (0.47-0.61)	<0.001	Moderate	99.9	42.5	87.8	98.9

Level of agreement and diagnostic accuracy are presented as κ-value (95% CI) and%, respectively. *p-values (approx. sig.). Chi-square test was used to compare the level of agreement between two different definitions of MetS.

## Discussion

Irrespective of the defining criteria, our study revealed a very high prevalence of MetS in Nepalese type 2 diabetic patients, far higher than those reported in the general population of Nepal and elsewhere [14,15,26-28]. Among our diabetic patients, the highest prevalence rate was estimated by Harmonized criteria (crude: 91.6%, age adjusted: 80.3%) followed by NCEP ATPIII (crude: 83.0%, age adjusted: 73.9%), WHO (crude: 81.1%, age adjusted: 69.9%) and IDF criteria (crude: 80.5%, age adjusted: 66.8%), respectively. These seemingly different prevalence rates arise due to the different cut-off points and sets of criteria used by these four different definitions. The performance of Harmonized criteria was the highest due to the removal of central obesity as an obligatory component and inclusion of any three of the five criteria present. This definition was introduced very recently in 2009 to bring the harmony in the several existing definitions of the MetS [8]. Studies that have used this definition also reported very high prevalence of MetS confirming its improved performance in other diabetic population too [29,30]. Because of the very high cut-off points for waist circumference, NCEP definition could identify relatively low number of patients with central obesity. Similar studies conducted in other subset of Nepalese diabetic population have also reported relatively lower prevalence using this definition [17,18]. However, there was little effect on the total prevalence rate due to freedom of including any three components. The high prevalence of MetS among our patients was not surprising as they were suffering from type 2 diabetes which itself was an entity of the MetS. Several studies around the globe have reported very high prevalence of MetS in type 2 diabetic patients irrespective of the definitions used, ethnicity and geographical area highlighting the common etiology of MetS [17,29-31].

The gender distribution of the prevalence differed in our subjects when based on NCEP ATP III, IDF and Harmonized definitions, with higher prevalence in females. However, no such difference was noted for the prevalence estimated by WHO definition. Our female patients were suffering from diabetes and hypertension for longer duration, had relatively poor glycemic control and were more obese and dyslipidemic than their male counterpart which might explain why they have increased prevalence of MetS. The less apparent gender difference in prevalence by WHO definition might be due to narrow differences in the sex–specific cut-off values of waist-hip ratio and HDL-cholesterol as they were the more prevalent components of MetS in our patients. It is possible that female patients of our study sought medical treatment later in the disease than men due to less access to finances and lack of awareness and self-determination about their own health status. The prevalence of MetS sharply increased with the age in both men and women and remained highest in 50–69 years age range. This is expected because predisposition of MetS in both men and women is strongly favored by age related processes such as gradual decrease in the basal metabolic rate, decreased growth hormone secretion, hypogonadism, stress induced hypercortisolism, abdominal fat deposition and concomitant insulin resistance [1]. The sharp decline of the prevalence at very high age group, on the other hand, might be due to increased frequency of death of individuals who were most susceptible to obesity related mortality such as coronary artery diseases and cerebrovascular events [26,32].

Analysis of prevalence according to the place of residence revealed that male patients living in urban areas had the much higher prevalence according to WHO and NCEP ATPIII definitions while the opposite was true according to NCEP ATPIII, IDF and Harmonized definitions. The gender distribution of MetS within patients residing in villages was also significant, with higher prevalence in females by all four definitions. Similarly, there was a significant difference in the prevalence between females residing in urban and village areas. Females residing in village area had higher prevalence than the urban females by all four definitions, although the difference was not significant for the WHO defined prevalence. We also analyzed distribution of prevalence according to ethnicity and occupational activity of the patients. Nepalese society is mainly agrarian in nature and a mixture of two major ethnic groups: Indo-Aryans and Mongoloids. Indo-Aryans are basically Hindus and are further divided into several social caste systems such as Brahmins, Chhetris, Vaishyas and Shudras or Dalits (Kami, Damai, Sarki, Gandharva etc.), while Mongoloids, although not divided into castes, have several tribes and lineages [33]. Analysis based on ethnicity revealed that total prevalence was the highest among Mongol, Dalit and Newar patients. Dalit female patients had consistently higher prevalence of MetS by all four definitions. In addition, prevalence was also highest among Chhetri and Newar female patients according to IDF and Harmonized definitions. However, it was highest among Mongoloid male patients by WHO, Dalit patients by NCEP ATPIII and Harmonized and Newar patients by IDF definitions. Likewise, prevalence was higher among patients who were associated with agriculture and particularly among males. In females, prevalence was higher among those who were involved either in business or office job. It has been established by several studies that low socioeconomic status, urban habitat, illiteracy, blue collar occupation and certain ethnicity are strongly associated with increased prevalence of MetS [28,34-36]. Majority of our patients associated with agriculture profession were residing in the village area and were either illiterate or moderately educated and belonged to the family of low socioeconomic status. The reasons for increased prevalence of MetS in urban patients were seemed to be sedentary life-style due to their involvement in business and office jobs, increased intake of calorie rich foods and android type central obesity.

We found substantial level of agreement between NCEP ATPIII and Harmonized definitions. NCEP ATPIII definition missed out only one diabetic patient having MetS according to Harmonized definition. This high level of concordance is not surprising given their identical criteria except the waist circumference. The second highest agreement was observed between NCEP ATPIII and WHO definitions and the lowest was observed between IDF and WHO definitions. The agreement was only fair between WHO and harmonized definitions. Almost similar pattern of agreement has been found between these pairs of definitions when used for other diabetic population [30,37]. From these observations, we can conclude that WHO, NCEP ATPIII and Harmonized definitions can identify patients with higher degree of insulin resistance and increased risk of cardiovascular diseases, but IDF definition can identify additional patients, not identified by the earlier definitions. These additional patients were those who are at increased risk of future CVDs but have lesser degree of insulin resistance. Among the four definitions used, harmonized definition was found to be the most sensitive while NCEP ATP III and IDF definitions were found to be most specific in identifying the cases of MetS.

Majority of our patients had a cluster of four components of MetS included by all four definitions: central obesity, high serum TG, low serum HDL-C and hypertension. This type of clustering is a commonly observed phenomenon among type 2 diabetic patients which significantly increases the risk of CVDs [13,38]. We found central obesity as the most prevalent component according to WHO and IDF criteria and decreased HDL-cholesterol according to NCEP and Harmonized criteria. The lower prevalence of central obesity according to the NCEP ATP III criteria must be due to its relatively higher cut-off values for waist circumference and that makes it less applicable criterion to the Nepalese population because of their smaller body sizes. Prevalence of BMI, central obesity and low HDL-C was significantly higher in females. South Asians, which also includes Nepali population, are shown to have increased visceral fat, central obesity, dyslipidemia and insulin resistance even at younger age group compared to their Western counterparts which predisposes them to very high risk of MetS, type 2 DM and cardiovascular diseases than any other population in the world [39]. In order to address these issues, WHO and IDF have set lower cut-off points for the BMI, waist circumference and waist hip ratio for South Asians [7,20]. It may thus be argued that central obesity and decreased HDL level can be used as the strong diagnostic markers of MetS also in Nepalese population. Hypertension, on the other hand, was found to be the least prevalent component in our study patients according to all four criteria and no significant relation was found with gender except for the prevalence estimated by NCEP ATP III definition. These findings are consistent with the reports of earlier studies conducted in Nepal [15,17,18] but conflicting with many other international studies [30,37,40]. In our study, patients with MetS were found to be older, more dyslipidemic, obese and hypertensive for longer duration than those without MetS which was quite expected and supported by many other studie [29,40]. These patients, thus, bore very high cardiometabolic risk even when compared to their diabetic counterparts without MetS [41].

Our study was a cross-sectional study conducted among uncomplicated type 2 diabetic patients attending a tertiary care teaching hospital located in the Western Development Region of Nepal. It enrolled mainly those patients who hailed from the different districts of this region of Nepal and therefore, may not necessarily represent entire Nepalese diabetic population. This study also did not cover the effect of treatment in the variation of MetS prevalence among the study patients. Despite these limitations, our study is the first of its kind in Nepal and reports the age adjusted prevalence rates of MetS among type 2 diabetics using four most used defining criteria. It also analyzes the prevalence rates according to the age-group, gender, place of residence, occupations, ethnicity and smoking and dietary habits of the study patients and describes the relative similarities and differences of the definitions used in identifying the cases of MetS. Moreover, it contributes to the mapping of epidemiology of MetS in Nepal and serves as comparable baseline data for health policy makers and researchers.

## Conclusion

Our study highlights the alarmingly high prevalence of MetS and the increased risk of strokes and cardiovascular diseases among Nepalese type 2 diabetic patients. It also suggests that Harmonized and NCEP ATP III definitions are better than WHO and IDF definitions in identifying the cases of MetS among Nepalese diabetic patients. We expect that these finding will prompt the concerned authorities of Nepal to formulate strategies to prevent and delay the onset of future complications among the diabetic patients. These strategies might include launching aggressive health education programs to increase the public awareness about the preventive measures and negative consequences of MetS and type 2 DM, investing more resources on the health care services making it more accessible to the general public and minimizing the individual risk factors by active therapeutic intervention in already affected individuals.

## Abbreviations

AHA: American Heart Association
BMI: Body mass index
BP: Blood pressure
CVDs: Cardiovascular diseases
DBP: Diastolic blood pressure
DM: Diabetes mellitus
FPG: Fasting plasma glucose
HDL-C: High density lipoprotein cholesterol
HTN: Hypertension
IDF: International Diabetes Federation
IAS: International Atherosclerosis Society
IASO: International Association for the Study of Obesity
IFG: Impaired fasting glucose
IGT: Impaired glucose tolerance
IR: Insulin resistance
LDL-C: Low density lipoprotein cholesterol
MetS: Metabolic syndrome
NCEP ATPIII: National Cholesterol Education Program Adult Treatment Panel III
NHLBI: National Heart Lung and Blood Institute
NPV: negative predictive value
PPV: Positive predictive value
PMG: Post meal glucose
Rx: Under medical treatment
SBP: Systolic blood pressure
TC: Total cholesterol
TG: Triglycerides
VLDL: Very low density lipoprotein
WC: Waist circumference
WHF: World Heart Federation
WHO: World Health Organization
WHR: Waist hip ratio
